# Deep learning for the detection of anatomical tissue structures and neoplasms of the skin on scanned histopathological tissue sections

**DOI:** 10.3389/fonc.2022.1022967

**Published:** 2022-11-22

**Authors:** Katharina Kriegsmann, Frithjof Lobers, Christiane Zgorzelski, Jörg Kriegsmann, Charlotte Janßen, Rolf Rüdinger Meliß, Thomas Muley, Ulrich Sack, Georg Steinbuss, Mark Kriegsmann

**Affiliations:** ^1^ Department of Hematology, Oncology and Rheumatology, Heidelberg University, Heidelberg, Germany; ^2^ Department of Clinical Immunology, Medical Faculty, University of Leipzig, Leipzig, Germany; ^3^ Institute of Pathology, Heidelberg University, Heidelberg, Germany; ^4^ MVZ Histology, Cytology and Molecular Diagnostics Trier, Trier, Germany; ^5^ Proteopath Trier, Trier, Germany; ^6^ Center for Industrial Mathematics (ZeTeM), University of Bremen, Bremen, Germany; ^7^ Institute for Dermatopathology, Hannover, Germany; ^8^ Translational Lung Research Centre (TLRC) Heidelberg, Member of the German Centre for Lung Research (DZL), Heidelberg, Germany

**Keywords:** deep learning, pathology, artificial intelligence, dermatopathology, digital pathology, deep learning - artificial neural network

## Abstract

Basal cell carcinoma (BCC), squamous cell carcinoma (SqCC) and melanoma are among the most common cancer types. Correct diagnosis based on histological evaluation after biopsy or excision is paramount for adequate therapy stratification. Deep learning on histological slides has been suggested to complement and improve routine diagnostics, but publicly available curated and annotated data and usable models trained to distinguish common skin tumors are rare and often lack heterogeneous non-tumor categories. A total of 16 classes from 386 cases were manually annotated on scanned histological slides, 129,364 100 x 100 µm (~395 x 395 px) image tiles were extracted and split into a training, validation and test set. An EfficientV2 neuronal network was trained and optimized to classify image categories. Cross entropy loss, balanced accuracy and Matthews correlation coefficient were used for model evaluation. Image and patient data were assessed with confusion matrices. Application of the model to an external set of whole slides facilitated localization of melanoma and non-tumor tissue. Automated differentiation of BCC, SqCC, melanoma, naevi and non-tumor tissue structures was possible, and a high diagnostic accuracy was achieved in the validation (98%) and test (97%) set. In summary, we provide a curated dataset including the most common neoplasms of the skin and various anatomical compartments to enable researchers to train, validate and improve deep learning models. Automated classification of skin tumors by deep learning techniques is possible with high accuracy, facilitates tumor localization and has the potential to support and improve routine diagnostics.

## 1 Introduction

Skin cancer is the most common cancer type in the United States (1). Patient management and prognosis is variable and depends on the entity, molecular changes, as well as on the clinical stage at the time of diagnosis (2). Skin cancer is a highly heterogeneous group composed of non-melanotic and melanotic neoplasms (3). Among the non-melanotic neoplasms, basal cell carcinoma (BCC) and squamous cell carcinomas (SqCC) are the most common (4) and usually well treatable. Despite major advances in treatment, most deaths from skin cancer are still due to melanoma (5). Thus, the correct diagnosis is paramount for treatment selection and prognosis.

Currently, the diagnosis of different cutaneous tumor types is based on physical examination, dermatoscopy and ultimately histological evaluation of an excision specimen. While reliable diagnosis can be made in a substantial number of tissue specimen on a regular standard stain alone, a significant subset of neoplastic skin lesions requires additional immunohistology and molecular studies for definite classification. In particular, the differentiation between BCC and SqCC, as well as between naevi and melanoma may be challenging. As the incidence of skin cancer is increasing, while the number of pathologists and dermato-pathologists is decreasing in many countries, the introduction of new methods to support skin tumor diagnostics is desirable (6).

The use of deep learning methods applied to clinical images, dermatoscopy images or scanned histopathological slides holds great promise to support cancer diagnostics in general (7, 8), and skin cancer diagnostics in particular (5, 9). In the past, the feasibility and potential to classify different diseases on scanned histological slides has been demonstrated for automated localization and diagnosis of melanoma (9, 10), the differentiation between naevi and melanoma (11), and the differentiation between basaloid, squamous, melanocytic and other skin tumors (12).

Major problems identified in previous studies for routine diagnostic application of such algorithms are: (i) no consideration of non-tumor skin categories or inclusion of only one non-tumor skin class, (ii) the need for manual annotation of the tissue region of interest prior to automated tumor classification, and (iii) non-availability of raw data images for validation purposes. In the current study, we therefore annotated major non-tumor anatomical tissue structures of the skin and major skin tumor categories and subsequently trained a convolutional neuronal network using an up-to-date workflow. We localized and categorized skin tumors on whole slides without prior annotation, validated our data on an external test set and provide all images and code to enable other researchers to improve and validate their data.

## 2 Methods

### 2.1 Patient data

Whole slides from patients with BCC (n = 93), SqCC (n = 100), naevi (n = 98) and melanoma (n = 87) were extracted from the archive of the Institute of Pathology, Heidelberg University, the MVZ for Histology, Cytology and Molecular Diagnostics Trier and the Institute for Dermatopathology Hannover. Diagnoses were made according to the World Health Organization (WHO) Classification of Skin Tumours (13). All slides with representative tumor regions were scanned using an automated slide scanner (Aperio AT2, Leica Biosystems, Nussloch, Germany) with 400 x magnification, as previously described (14). Image data were anonymized and are provided along with this manuscript (Link: https://heidata.uni-heidelberg.de/privateurl.xhtml?token=366931ac-50a2-43f9-880f-88d63e07d493). Moreover, an independent external dataset of melanoma whole slides was downloaded from the website of the Cancer Imaging Archive (CPTAC-CM) (15). After quality review 62 cases were included as an external test set, while 41 of these cases were melanoma and 21 were tumor-free skin. The analysis was approved by the local ethics committee of Heidelberg University.

### 2.2 Image data

**Figure 1:**
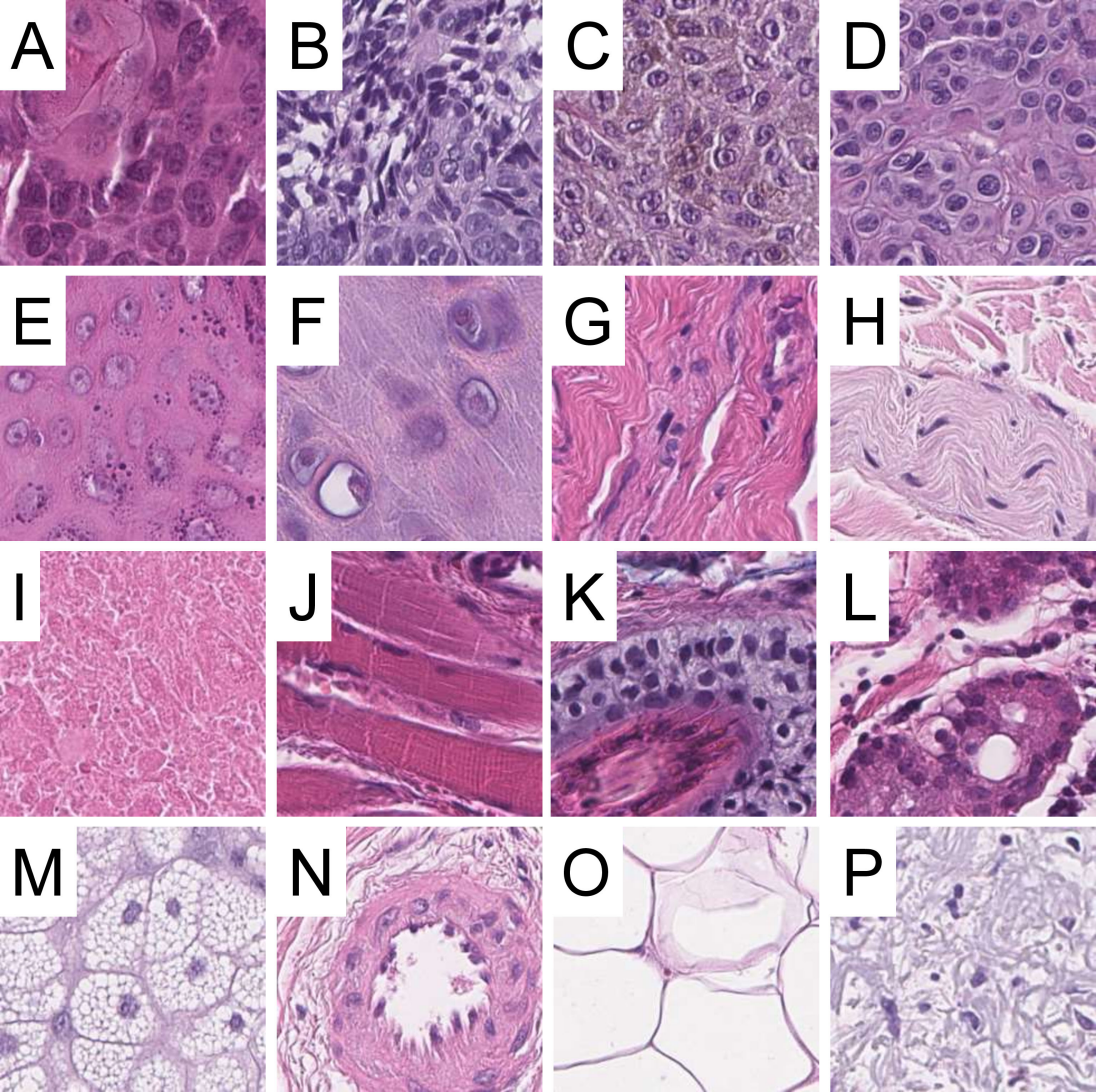
Examples of image patches included in the dataset. Squamous cell carcinoma **(A)**, basal cell carcinoma **(B)**, melanoma **(C)**, naevi **(D)**, epidermis **(E)**, chrondral tissue **(F)**, dermis **(G)**, nerves **(H)**, necrosis **(I)**, skeletal muscle **(J)**, hair follicles **(K)**, sweat glands/eccrine glands **(L)**, sebaceous glands **(M)**, vessels **(N)**, subcutis **(O)** and elastosis **(P)**. All images are 100 x 100 µm (395 x 395 px) in size, scale bar = 20 µm.

Scanned histopathological slides were imported into QuPath (16) (v.0.1.2, University of Edinburgh, Edinburgh, UK) and annotated (F.L. and M.K.) for the following 16 categories: chondral tissue, dermis, elastosis, epidermis, hair follicle, skeletal muscle, necrosis, nerves, sebaceous glands, subcutis, eccrine glands (sweat glands), vessels, BCC, SqCC, naevi and melanoma. Image patches 100 x 100 µm (~395 x 395 px) in size were generated in QuPath, extracted on the local hard drive and subsequently reviewed. Blurry images were deleted. Representative image patches are displayed in **Figure 1**.

### 2.3 Splitting of datasets

Images from patients were separated into a training, validation and test set. All image patches from one patient were used in only one of the respective sets. Since there are only three cases with elastosis, we assigned the case with most elastosis patches to the training, the case with the second most patches to the test and the remaining case to the validation set. All other cases were assigned randomly to one of the sets. The sets were not changed during the analyses. The splits by image patches and patients are displayed in **Table 1**.

**Table 1:** Number of image patches and patients in the training, validation and test set.

Class	Training, n (%)		Validation, n (%)		Test, n (%)	
	by patches	by patient	by patches	by patient	by patches	by patient
Chondral tissue	4442 (62)	9 (50)	743 (10)	2 (11)	1992 (28)	7 (39)
Dermis	15878 (70)	134 (69)	1857 (8)	20 (10)	4875 (22)	39 (20)
Elastosis	136 (65)	1 (33)	6 (3)	1 (33)	66 (32)	1 (33)
Epidermis	10419 (74)	130 (70)	1086 (8)	19 (10)	2613 (19)	36 (19)
Hair follicle	1437 (71)	104 (71)	250 (12)	15 (10)	325 (16)	27 (18)
Skeletal muscle	6159 (80)	47 (73)	904 (12)	7 (11)	669 (9)	10 (16)
Necrosis	1641 (54)	24 (67)	468 (15)	5 (14)	924 (30)	7 (19)
Nerves	1201 (64)	93 (68)	219 (12)	13 (10)	464 (25)	30 (22)
Sebaceous glands	7268 (67)	94 (69)	1074 (10)	13 (9)	2565 (24)	30 (22)
Subcutis	7370 (61)	64 (65)	1245 (10)	9 (9)	3438 (29)	26 (26)
Sweat glands	2533 (71)	94 (71)	220 (6)	11 (8)	818 (23)	27 (20)
Vessels	1068 (65)	109 (71)	136 (8)	14 (9)	439 (27)	31 (20)
BCC	6919 (78)	71 (76)	1063 (12)	12 (13)	941 (11)	10 (11)
SqCC	6793 (61)	61 (61)	919 (8)	10 (10)	3470 (31)	29 (29)
Naevi	7923 (75)	72 (73)	944 (9)	8 (8)	1762 (17)	18 (18)
Melanoma	7784 (67)	59 (68)	1220 (10)	9 (10)	2678 (23)	19 (22)

BCC, basal cell carcinoma; SqCC, squamous cell carcinoma.

### 2.4 Hard- and software

For training we used a p3.2xlarge instance from Amazon Web Services with a single V100 GPU while for inference we used a Lenovo P1 Gen 2 laptop. Further we used the Scientific Data Storage (SDS) service from Heidelberg University. Training and inference were performed using a singularity container image based on the TensorFlow Docker container image. For random augmentation we used the respective function in the image python module. The code is available at Link: https://heidata.uni-heidelberg.de/privateurl.xhtml?token=366931ac-50a2-43f9-880f-88d63e07d493.

### 2.5 Training and validation of models

Each model is based on the EfficientNetV2 architecture (17), was trained for a total of 30 epochs and with a learning rate of either 0.01, 0.001 or 0.001. We used a batch size of 64 for the smaller S models and 32 for the medium sized M models (cf. Larger Model). We used the AMSGrad optimizer (a variant of the Adam optimizer (18) with β1 = 0.9, β2 = 0.999 and ϵ^ = 1.0∗10^−7^). During training the data was sampled such that there was no class imbalance. We used random augmentation (19) with n=2 to reduce overfitting (M is different for the models, see below). Each model configuration (a set of model hyperparameters, e.g. the learning rate) was trained three times to account for the randomness involved model training (e.g. the random weights initialization). We first trained a few models to find a good learning rate, then tested if a learning rate schedule, progressive training (17) or a larger model improved prediction quality.

Hyperparameters for the initial models were as follows: image input size of 300 x 300 px, a dropout of 0.3 and M=15 for random augmentation. Using the learning rate scheduler, the learning rate was linearly increased from 0 to its base value (e.g. 0.01) for the first five epochs as in the original EfficientNetV2 publication (so called warm-up) (17) and subsequently exponentially decayed with a rate of 0.97. In progressive training the training set was split in different stages. In each stage, i.e. after a certain number of epochs, size of input images and regularization (such as dropout) were increased. The aim of progressive training was to improve training speed by using smaller image sizes on early epochs. Hyperparameters for the first 15 epochs were: image size of 128 x 128 px, top dropout 0.1 and M = 5 for random augmentation. Hyperparameters for the last 15 epochs were: image size of 300 x 300 px, top dropout 0.3 and M = 15 for random augmentation. For the larger model, we used the EfficientNetV2 M instead of the EfficientNetV2 S. We had to decrease the batch size to 32 such that the model and data fitted into the GPU memory. For all EfficientNetV2 M models, progressive learning was applied with the following hyperparameters: for the first 15 epochs: image size of 128 x 128 px, top dropout 0.1 and M = 5 for random augmentation; for the last 15 epochs: image size of 380 x 380 px, top dropout 0.4 and M = 20 for random augmentation. All models were subsequently compared and the model with the best performance was selected.

For each training run we recorded cross entropy loss, balanced accuracy (BAC) and Matthews correlation coefficient (MCC) for training as well as validation data (20). While we recorded and display all three metrics, we used MCC to select the best model. The validation set results were evaluated using confusion matrices. To visualize the proximity of the different classes, the last convolutional layer after the last pooling operation of the validation data was subjected to dimension reduction using uniform manifold approximation and projection (UMAP) computed *via* the Python package umap-learn.

### 2.6 Evaluation of the test set and on the external set

The best performing model (cf. Section 2.5) was applied to the test set. As all data were scanned with one scanner system and all cases were derived from institutions in Germany, we additionally applied our algorithm on an external set of melanomas where preprocessing of the tissue and most scanning conditions were unknown and staining properties were different. As the external test data had a high burden of artefacts, all slides were manually reviewed and only cases that passed a quality control were used for further analysis. However, remaining slides still had a relatively high burden of artefacts such as blurry areas, tissue tears, variation in tissue thickness, dust particles, high amount of necrosis and overall low tissue and staining quality. In addition, the magnification of the external slides was 200x which was different compared to the magnification of our data which was 400x. We decided to still test our algorithm on this set to evaluate suboptimal input data.

## 3 Results

### 3.1 Model training and optimization

In total 129,364 image tiles from 386 cases were used for training. Within all broader categories of models, a learning rate of 0.001 seemed to perform best regarding MCC on the validation data. The initial models (**Supplementary Figure 1**), the models trained with a learning rate scheduler (**Supplementary Figure 2**), models trained using progressive learning (**Supplementary Figure 3**) and larger models (**Supplementary Figure 4**) are depicted in the supplementary materials.

**Figure 2:**
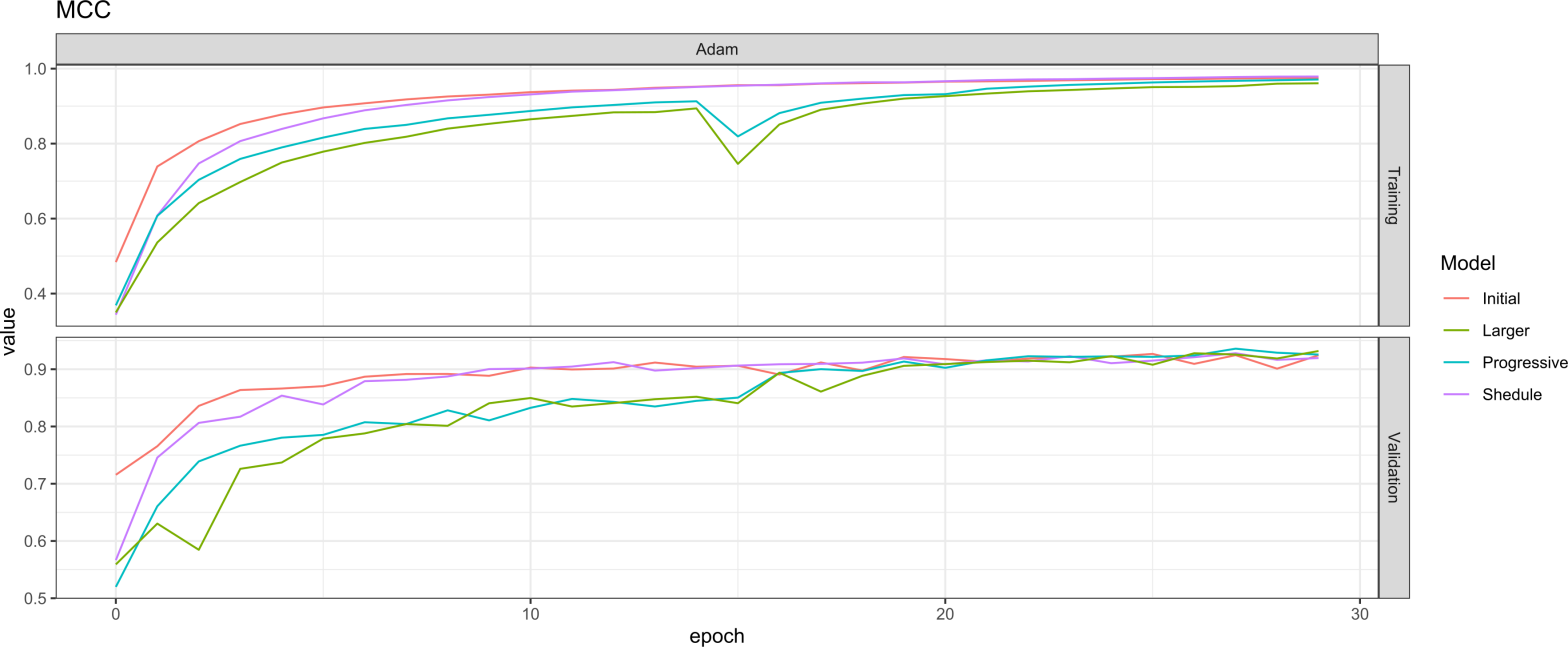
Matthews correlation coefficient (MCC) for the best models. MCC increases with the initial model, the model with learning rate scheduler, the model with progressive training and the larger model across the training process in the training as well as in the validation set. Models which used progressive learning show a drop at epoch 15 which corresponds to the changing hyperparameter settings at this point of time. MCC, Matthews correlation coefficient.

We compared the best performing models to choose the final model (**Figure 2**). The best performing model based on either loss, BAC or MCC are shown in **Table 2**. We decided for the model performing best in terms of MCC which was trained using the following configuration: EfficientNetV2 S, batch size of 64, progressive learning and learning rate of 0.001.

**Table 2:** The three best models after training based on loss, balanced accuracy and Matthews correlation coefficient.

Metric	Model ID	Epoch	Training			Validation		
			loss	BAC	MCC	loss	BAC	MCC
Loss	19	27	0.08955	0.96953	0.96750	0.18650	0.91650	0.93590
BAC	20	28	0.08871	0.96920	0.96760	0.18998	0.94276	0.93178
MCC	19	27	0.08955	0.96953	0.96750	0.18650	0.91650	0.93590

BAC, balanced accuracy; ID, Identifier; MCC, Matthews correlation coefficient.

### 3.2 Evaluation on the validation data

**Figure 3:**
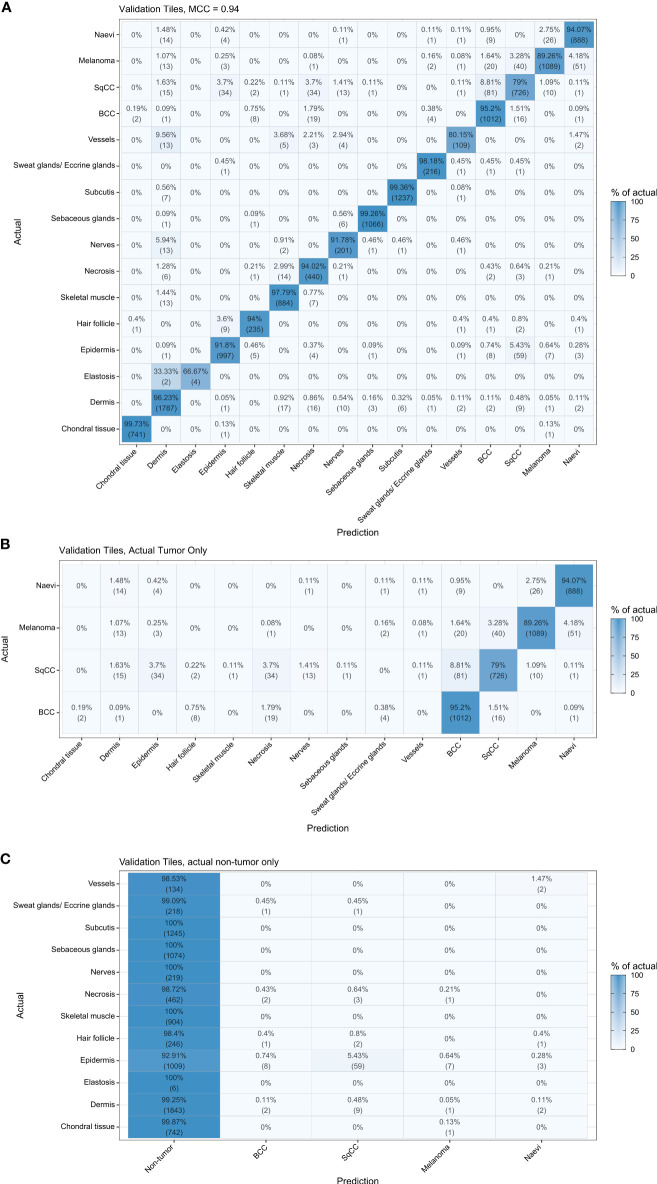
Confusion matrix of the validation set. High concordance between actual and predicted classes can be observed. The algorithm shows a higher rate of misclassification of elastosis and vessels with dermis, which can be explained since elastotic changes and vessels are commonly observed in the dermis **(A)**. In tumor categories **(B)** a higher rate of misclassifications was observed for squamous cell carcinomas that were predicted as basal cell carcinoma. The misclassification of non-tumor categories as tumor was rare but observed with epidermis, misclassified as squamous cell carcinoma **(C)**. BCC, basal cell carcinoma; SqCC, squamous cell carcinoma.

A confusion matrix shows generally high concordance between actual and predicted classes on the validation set based on image patches (**Figure 3A**). Within the tumor category, naevus was mostly misclassified as melanoma (2.75%) and vice versa (4.18%). Moreover, SqCC was most commonly misclassified as BCC (8.81%) and vice versa (1.51%). Among the non-tumor categories, the classes with misclassifications of > 5% were elastosis (33.33%), vessels (9.56%) and nerves (5.94%), that were misclassified as dermis. The non-tumor category that had highest misclassification rates was epidermis which was misclassified as SqCC (5.43%) and vice versa (3.70%, **Figures 3B, C**).

**Figure 4:**
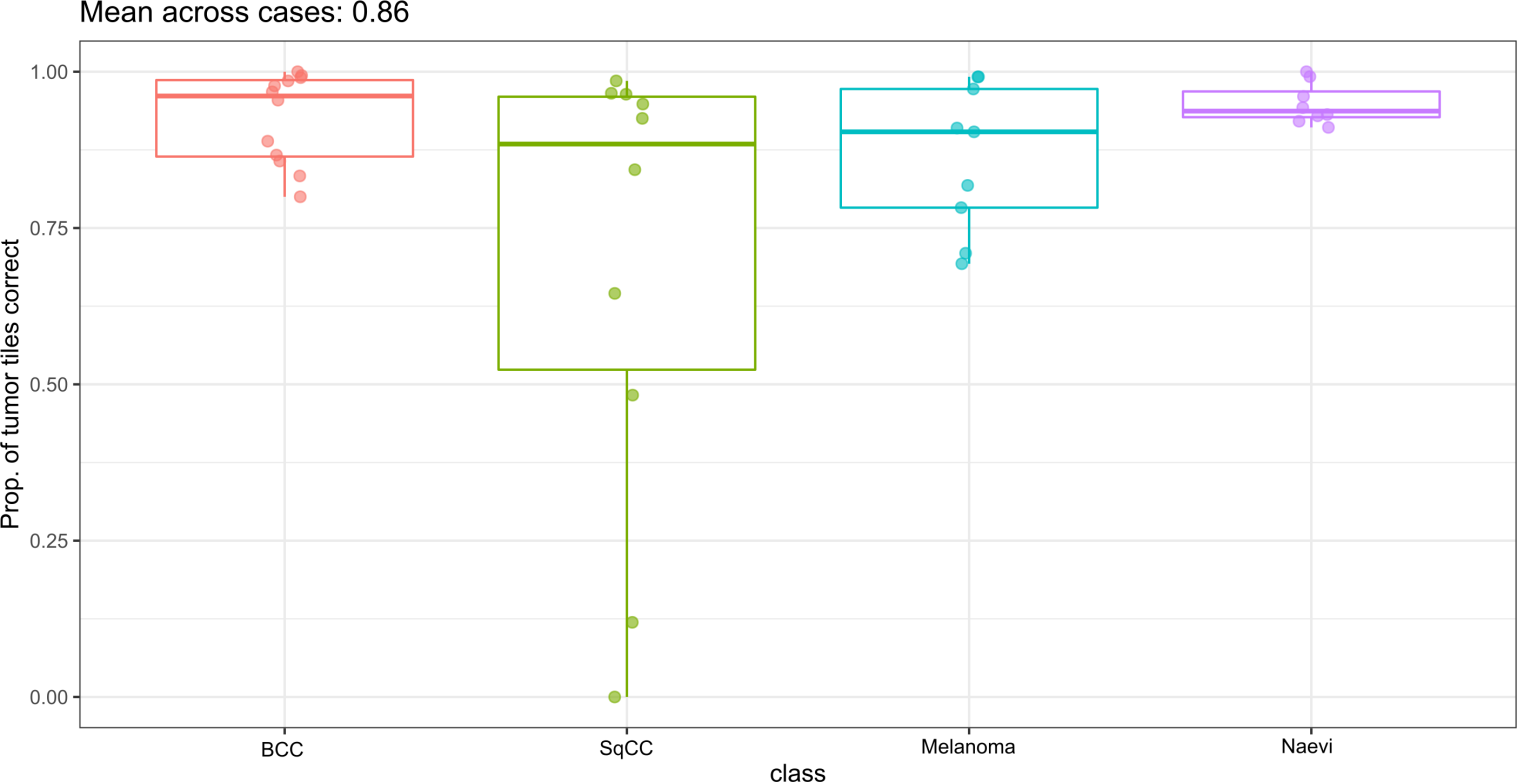
Proportion of tumor image tiles that was correctly classified on patient level in the validation set. Most tumors were correctly classified on patient level. Two patients with squamous cell carcinomas were misclassified. BCC, basal cell carcinoma; SqCC, squamous cell carcinoma.

To render a final diagnosis on a whole slide in the routine diagnostic scenario, the image patch-based result may not be very informative. Thus, we evaluated the proportion of the tumor image tiles that were correct on the case level (**Figure 4**). Overall, only two out of 81 (2.5%) tumor patients had very low proportions of image tiles that voted for the correct diagnosis. Interestingly, in both cases the correct diagnosis was squamous cell carcinoma. When examining both cases in detail (**Supplementary Figure 5**), one case would have finally been misclassified based on a majority vote for the final tumor class. In the other case, misclassifications in the non-tumor category would have luckily led to the correct diagnosis based on a majority vote for the final diagnosis, which would result in a final diagnostic accuracy of 98.7% (80/81).

### 3.3 Evaluation on the test data

For test data a confusion matrix showed a high degree of concordance between actual and predicted classes based on image patches (**Supplementary Figure 6**). The test data generally exhibited the same misclassifications as described on the validation data. Likewise, on the case level (**Supplementary Figure 7**) the diagnostic accuracy regarding the tumor classes was high with 74 out of 76 (97.4%) correct classifications. The respective confusion matrices of the two misclassified cases are provided in **Supplementary Figure 8**. When non-tumor and tumor classes were taken together, 73 out of 76 (96.0%) of cases were correctly classified. Among the three cases with a wrong classification result, one case was a SqCC misclassified as BCC and two cases were melanoma misclassified as SqCC.

### 3.4 Visualization of the resemblance of the images

**Figure 5:**
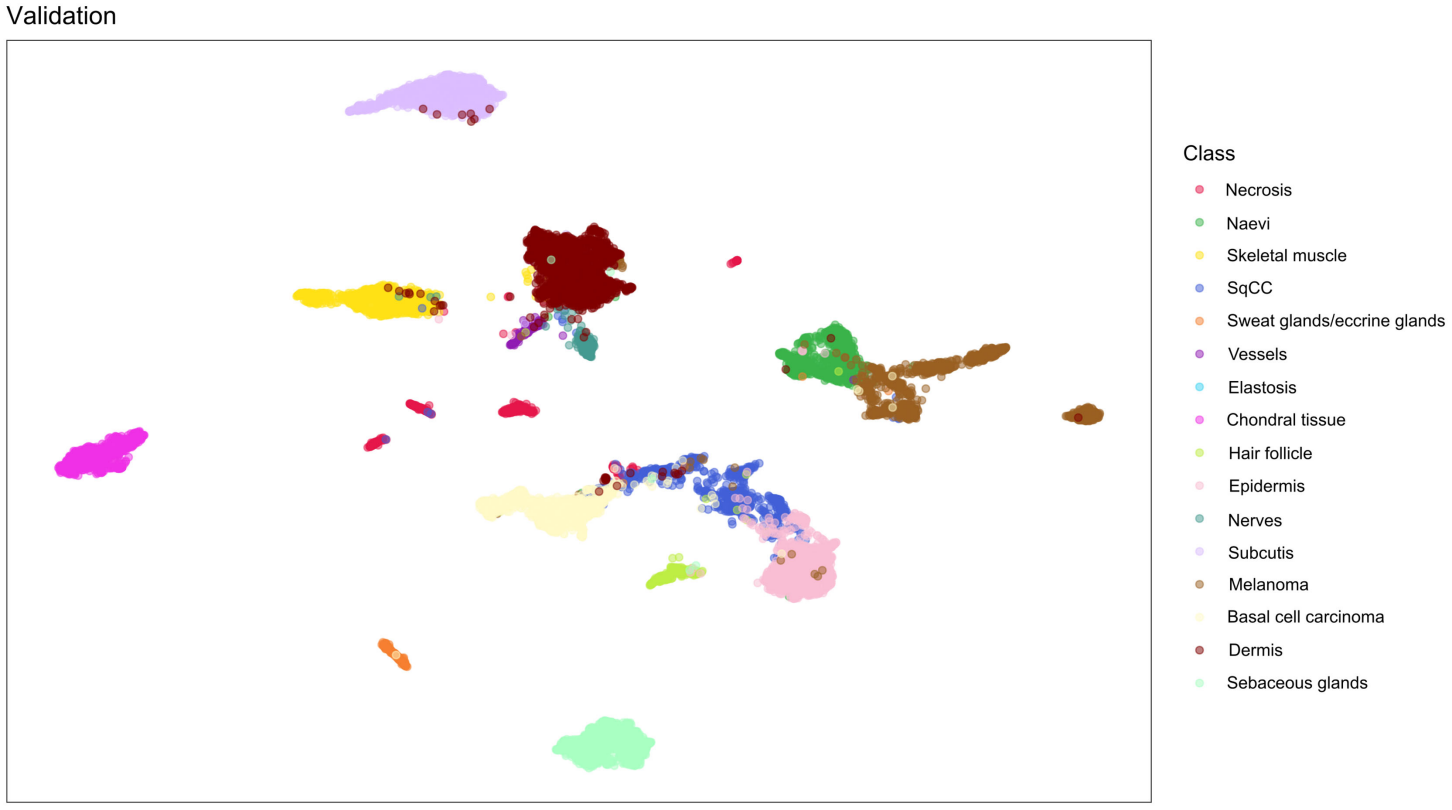
Dimension reduction using uniform manifold approximation and projection based on the last convolutional layer after the last pooling operation of the validation data. Close proximity of image classes that resemble each other morphologically such as melanocytic tumors can be observed. On the other hand, image categories that are morphologically very different such as skeletal muscle, sebaceous glands or chrondral tissue show distinct clusters. BCC, basal cell carcinoma; SqCC, squamous cell carcinoma.

To visualize the resemblance of the images, the output of last convolutional layer after the final pooling operation was subjected to dimension reduction. A UMAP diagram confirms that classes that are very different morphologically such as skeletal muscle, sebaceous glands and chondral tissue, are separated clearly from other image categories. On the other hand, melanocytic lesions such as naevi and melanoma show proximity and also some overlap. UMAP diagram of validation data (**Figure 5**) and test data (**Supplementary Figure 9**) are displayed.

### 3.5 Evaluation on the external test set

A total of 62 external slides passed the initial quality control. Quality issues were noted even in the remaining cases that passed the quality control. Examples of quality issues of the external test set are provided in **Supplementary Figure 10**. Of the 41 melanoma cases, 32 were predicted as melanoma (78%), 7 cases were predicted as BCC (17%), one case (2%) was predicted as SqCC and naevus, respectively. Non-tumor skin cases had generally a proportion of tiles predicted as tumor <5% of all image tiles and were rather randomly distributed throughout the whole slide.

## 4 Discussion

In the past, deep learning techniques have been shown to support diagnosis and prognosis prediction in many neoplastic and non-neoplastic diseases, such as but not limited to prostate cancer (21), lung cancer (22), breast cancer (23), pancreatic cancer (8), colon cancer (24), skin cancer (25, 26), cancer of unknown primary (27), or scoring of fibrosis or fat in non-neoplastic liver disease (28, 29).

In skin diseases, the technique has been mainly used in neoplastic diseases so far. While most reports focus on either melanocytic (9–11, 30) or non-melanocytic (26) lesions, data on both melanocytic and non-melanocytic lesions, non-tumor skin lesions or anatomical tissue structures are scarce (12, 26). Although there are honorable exceptions (31), most of the published studies on deep learning on histopathological slides do not make their annotated data, the full dataset of image patches and/or their code available. Our study narrows this gap by providing image data of 16 different classes including normal anatomical tissue structures, reactive solar elastosis and the most common neoplastic skin lesions. This complements the dataset from Thomas et al., who provided a publicly available comprehensive segmented dataset including 12 different classes for non-melanoma skin cancer and anatomical tissue structures. We hope our data will enable researchers to validate our and their results and to develop new methods for the application of deep learning to support pathologists and ultimately improve patient care. Of note, we believe it is necessary to avoid a common non-tumor skin category and to separate morphologically distinct classes during training, as the different anatomical tissue structures are morphologically highly heterogeneous, which has also been highlighted by a UMAP diagram in the current study. The introduction of non-tumor skin categories will also enable automated classification of whole slides, without prior annotation of the tumor area, which is important to achieve a workflow that is faster than the current analog setting, although we are aware of recent developments that may overcome this issue (32). Additionally, the identification of anatomical tissue structures may be suitable for automatic tissue orientation with subsequent automatic distance measurements of the tumor margin (26).

Technically, we have used the EfficientNetV2, which has achieved high top-1 and top-5 accuracies on the ImageNet reference dataset and is a modern and efficient alternative to larger and more computationally expensive architectures available (17). In the past other algorithms, specifically for segmentation or specific organ systems have been published (33, 34). The architecture used in this study has been successfully applied to histological images previously (35). We have successfully applied techniques like image augmentation of progressive learning that have been suggested to find a well performing model in the current study (17). To train a reliable deep learning model, a large number of images is usually necessary to account for technical and biological variation. In this regard, a higher number of patient samples is commonly preferred over a large number of images. The number of patients included for training, validation and testing in the current investigation is within the reported range of previous studies (9, 11, 26). Currently, there is no consensus on the minimum number of cases that should be included in a deep learning study. Algorithms that require manual annotation comprise often a much lower number of patients as compared to approaches that do not need manual annotation. The previously reported studies on deep learning on histopathological slides have included between dozens to >1000 cases per entity (32, 36). The largest manually annotated publicly available dataset on skin cancer subtypes comprises 290 whole slides. Our study includes a total of 16 classes from 386 manually annotated cases which is within the reported range. Thus, we think that our approach is valid although there is no firmly established standard to calculate sample sizes for deep learning studies. Our training and validation workflow included not only a training and validation set, but also an internal and an external test set. This strategy is regarded as good scientific practice (37). Although we tested only for melanoma and normal tissue in the external test set, we still believe that the approach is legit, as melanoma is by far the disease with the worst prognosis and because the external dataset exhibited severe artifacts and was therefore suitable to test for plausibility of our model.

Moreover, we show the application of our model that was trained on small image patches, to whole slides, which allows rapid identification of anatomical tissue structures and neoplastic lesions. This may result in a faster and more focused review of tissue sections in the routine diagnostic setting, as regions of interest are highlighted. Moreover, areas with high tumor cell content are automatically highlighted for potential dissection and subsequent molecular analyses.

The performance of our model on the image level is within the range that has been reported by others (9–11). Our model had weaknesses in the distinction of BCC and SqCC, naevi and melanoma, epidermis and SqCC and elastosis, vessels and nerves with dermis. All these misclassifications can be explained: First, BCC may exhibit squamous differentiation and SqCC may look basaloid. Second, the distinction of naevi and melanoma may be challenging and the criteria for correct classification include the assessment on low magnification power. As we provided only high magnification power images for training and cytology not always resolve the differential, some misclassified images were expected. Third, SqCC is derived from the epidermal compartment and both classes are therefore composed of the same cells. Especially in highly differentiated SqCC the morphological difference to epidermis may be minimal to absent on high-power. Fourth, elastosis, vessels and nerves are all located within the collagenous dermal skin compartment. Thus, the classification of an image containing e.g. vessels as dermis, is not necessarily a misclassification.

Although, our algorithm showed a decreased performance of 78% for melanoma and 84% in all whole slides on the external test set considering a majority vote, we still believe that the performance is reasonably good and support the use of our classifier for research, given the rather poor overall quality of this external cohort.

The limitations of our study include the number of patients and the number of different entities included to train the model. The full morphological spectrum of BCC, SqCC, naevi and melanoma cannot fully be displayed with the number of patients included in this study. Likewise, the number of cutaneous neoplasms is by far larger as the four most common tumor types included in this study and entities not trained, cannot be identified by the classifier. Based on the above-mentioned limitations the application of such a deep learning model can only be a diagnostic supplement and should always be conducted under supervision of an expert pathologist or dermatopathologist, to avoid potentially harmful consequences for patients.

In summary, we show that the automated identification and classification of common skin tumors is possible by deep learning on scanned histological tissue sections and may contribute to an efficient workflow in routine diagnostics.

## Data availability statement

The datasets for this study can be found here: https://doi.org/10.11588/data/7QCR8S and here: https://wiki.cancerimagingarchive.net/pages/viewpage.action?pageId=33948224. The code to conduct the analysis can be found here: https://doi.org/10.11588/data/7QCR8S.

## Ethics statement

The study was approved by the Ethics committee of Heidelberg University, #315/20. Written informed consent for participation was not required for this study in accordance with the national legislation and the institutional requirements.

## Author contributions

Conception and design of the study, MK and KK. Data acquisition, FL. Data curation, MK, CZ, FL, JK, and RRM. Data analysis, KK, FL, CZ, GS, and MK. Visualization, KK, FL, GS, and MK. Financial support, KK, GS, JK, US, MK, and TM. Supervision, KK, JK, US, and MK. Manuscript draft, FL and MK. Review for important intellectual content, KK, FL, JK, CZ, CJ, RM, US, TM, GS, and MK. All authors contributed to the article and approved the submitted version

## Acknowledgments

The authors gratefully acknowledge the data storage service SDS@hd supported by the Ministry of Science, Research and the Arts Baden-Württemberg (MWK) and the German Research Foundation (DFG) through grant INST 35/1314-1 FUGG and INST 35/1503-1 FUGG. Data used in this publication (external test set) were generated by the National Cancer Institute Clinical Proteomic Tumor Analysis Consortium (CPTAC).

## Conflict of interest

The authors declare that the research was conducted in the absence of any commercial or financial relationships that could be construed as a potential conflict of interest.

## Publisher’s note

All claims expressed in this article are solely those of the authors and do not necessarily represent those of their affiliated organizations, or those of the publisher, the editors and the reviewers. Any product that may be evaluated in this article, or claim that may be made by its manufacturer, is not guaranteed or endorsed by the publisher.
