# Corrigendum: Deep learning for the detection of anatomical tissue structures and neoplasms of the skin on scanned histopathological tissue sections

**DOI:** 10.3389/fonc.2023.1201237

**Published:** 2023-05-18

**Authors:** Katharina Kriegsmann, Frithjof Lobers, Christiane Zgorzelski, Jörg Kriegsmann, Charlotte Janßen, Rolf Rüdinger Meliß, Thomas Muley, Ulrich Sack, Georg Steinbuss, Mark Kriegsmann

**Affiliations:** ^1^ Department of Hematology, Oncology and Rheumatology, Heidelberg University, Heidelberg, Germany; ^2^ Department of Clinical Immunology, Medical Faculty, University of Leipzig, Leipzig, Germany; ^3^ Institute of Pathology, Heidelberg University, Heidelberg, Germany; ^4^ MVZ Histology, Cytology and Molecular Diagnostics Trier, Trier, Germany; ^5^ Proteopath Trier, Trier, Germany; ^6^ Center for Industrial Mathematics (ZeTeM), University of Bremen, Bremen, Germany; ^7^ Institute for Dermatopathology, Hannover, Germany; ^8^ Translational Lung Research Centre (TLRC) Heidelberg, Member of the German Centre for Lung Research (DZL), Heidelberg, Germany

**Keywords:** deep learning, pathology, artificial intelligence, dermatopathology, digital pathology, deep learning - artificial neural network

**Incorrect Data Availability Statement**

In the published article, there was an error in the Data Availability statement. The links provided for the dataset and the code of the study were incorrect. The Data Availability statement was displayed as “The datasets for this study can be found here: https://heidata.uni-heidelberg.de/privateurl.xhtml?token=366931ac-50a2-43f9-880f-88d63e07d493 and here: https://wiki.cancerimagingarchive.net/pages/viewpage.action?pageId=33948224. The code to conduct the analysis can be found here: https://heidata.uni-heidelberg.de/privateurl.xhtml?token=366931ac-50a2-43f9-880f-88d63e07d493.”

The correct Data Availability statement appears below.

**Data availability statement**

The datasets for this study can be found here: https://doi.org/10.11588/data/7QCR8S and here: https://wiki.cancerimagingarchive.net/pages/viewpage.action?pageId=33948224. The code to conduct the analysis can be found here: https://doi.org/10.11588/data/7QCR8S.

The authors apologize for this error and state that this does not change the scientific conclusions of the article in any way. The original article has been updated.

**Missing Citation**

In the published article, 38. *Katharina Kriegsmann; Fritjof Lobers; Christiane Zgorzelski; Jörg Kriegsmann; Rolf Rüdinger Meliß; Ulrich Sack; Georg Steinbuss; Mark Kriegsmann, 2022, “Deep learning for the detection of anatomical tissue structures and neoplasms of the skin on scanned histopathological tissue sections [data]”*, https://doi.org/10.11588/data/7QCR8S *, heiDATA, V1*, was not cited in the article. The citation has now been inserted in Methods, Patient data, Paragraph 1, and should read:

“Whole slides from patients with BCC (n = 93), SqCC (n = 100), naevi (n = 98) and melanoma (n = 87) were extracted from the archive of the Institute of Pathology, Heidelberg University, the MVZ for Histology, Cytology and Molecular Diagnostics Trier and the Institute for Dermatopathology Hannover. Diagnoses were made according to the World Health Organization (WHO) Classification of Skin Tumours (13). All slides with representative tumor regions were scanned using an automated slide scanner (Aperio AT2, Leica Biosystems, Nussloch, Germany) with 400 x magnification, as previously described (14). Image data were anonymized and are provided along with this manuscript (38). Moreover, an independent external dataset of melanoma whole slides was downloaded from the website of the Cancer Imaging Archive (CPTAC-CM) (15). After quality review 62 cases were included as an external test set, while 41 of these cases were melanoma and 21 were tumor-free skin. The analysis was approved by the local ethics committee of Heidelberg University.”

The citation has also been inserted in Methods, Hard- and software, Paragraph 1, and should read:

“For training we used a p3.2xlarge instance from Amazon Web Services with a single V100 GPU while for inference we used a Lenovo P1 Gen 2 laptop. Further we used the Scientific Data Storage (SDS) service from Heidelberg University. Training and inference were performed using a singularity container image based on the TensorFlow Docker container image. For random augmentation we used the respective function in the image python module. The code is available at (38)”.

The authors apologize for this error and state that this does not change the scientific conclusions of the article in any way. The original article has been updated.

