# Supplementary material for: Deep learning for the detection of anatomical tissue structures and neoplasms of the skin on scanned histopathological tissue sections
Source: Front Oncol. 2022 Nov 22;12:1022967. doi: 10.3389/fonc.2022.1022967 (PMC9723465; doi:10.3389/fonc.2022.1022967)
Supplement: Supplementary file 1 [file DataSheet_1.pdf]

Supplementary Material

A

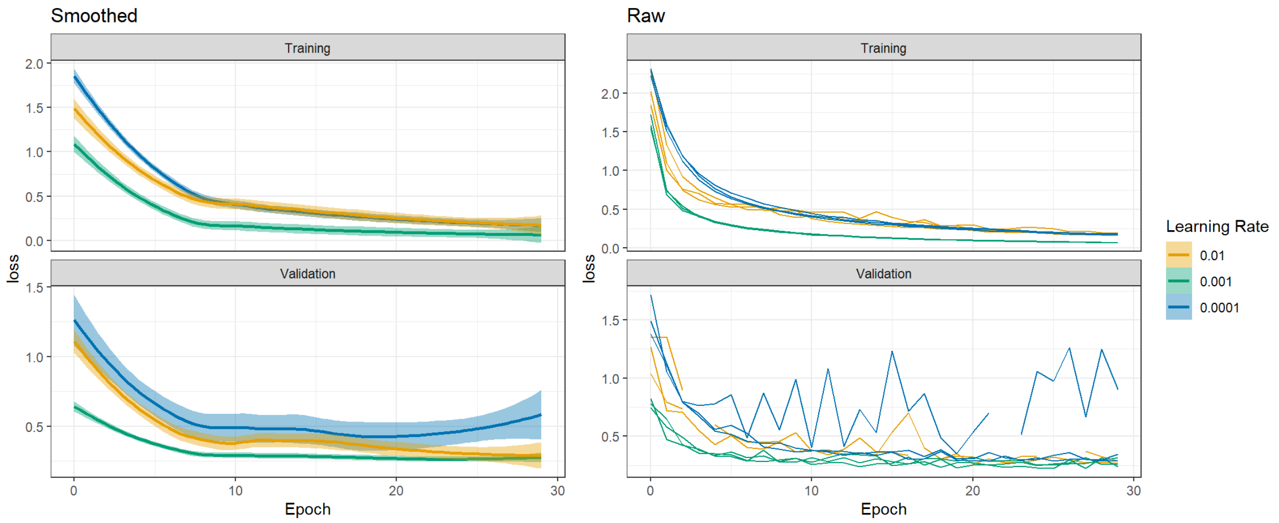

B

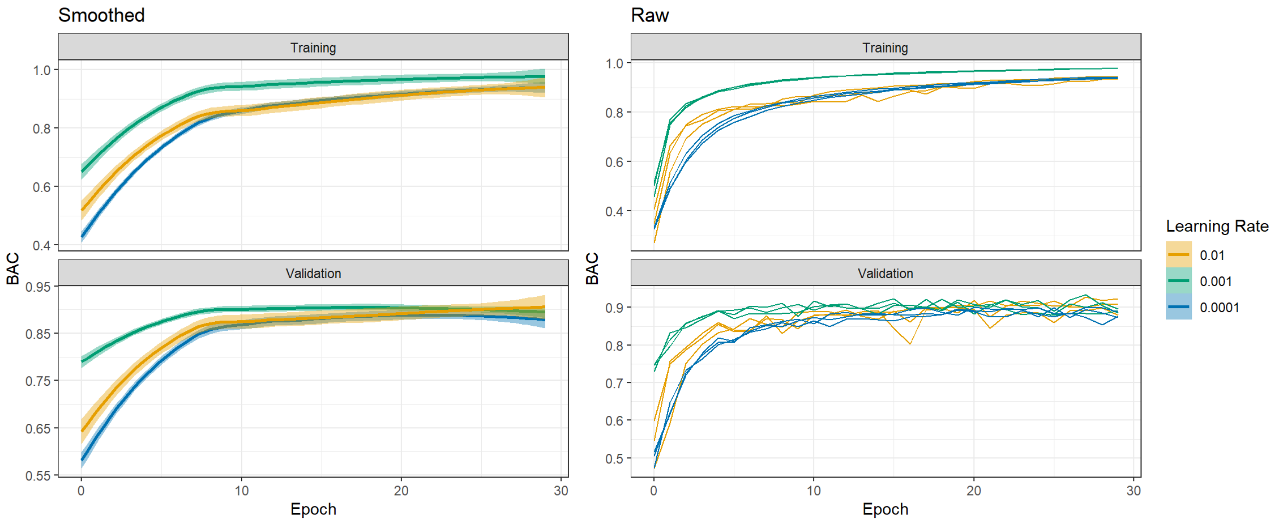

C

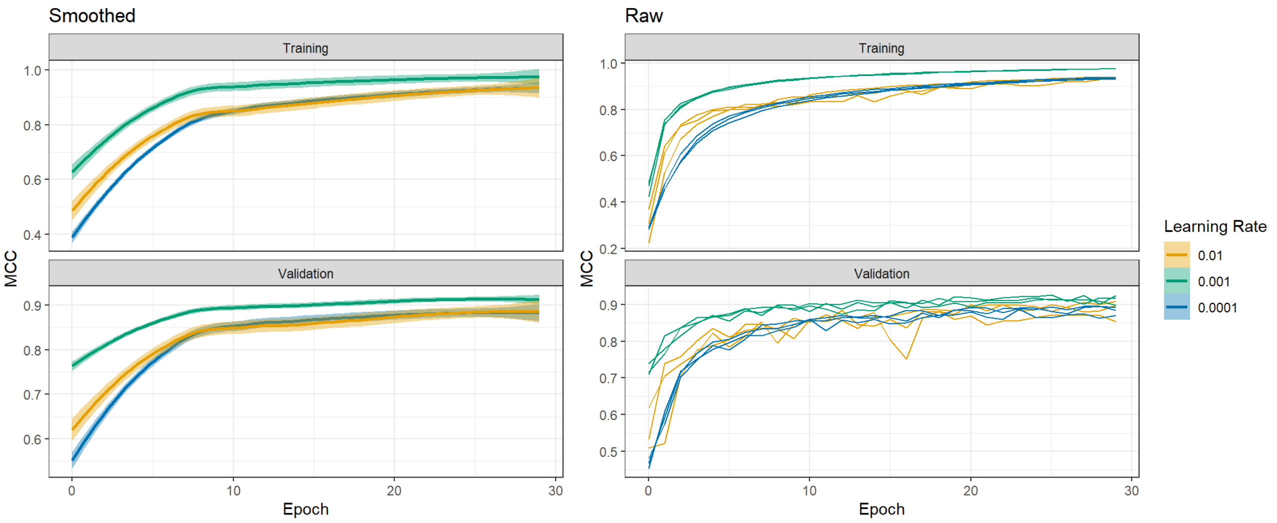

Supplemental Figure 1: Initial model. Cross entropy loss (A), balanced accuracy (B) and Matthews correlation coefficient (C) for training and validation data across all training epochs. The plots on the left show smoothed values for the three different iterations, while the plots on the right show the raw values.

A

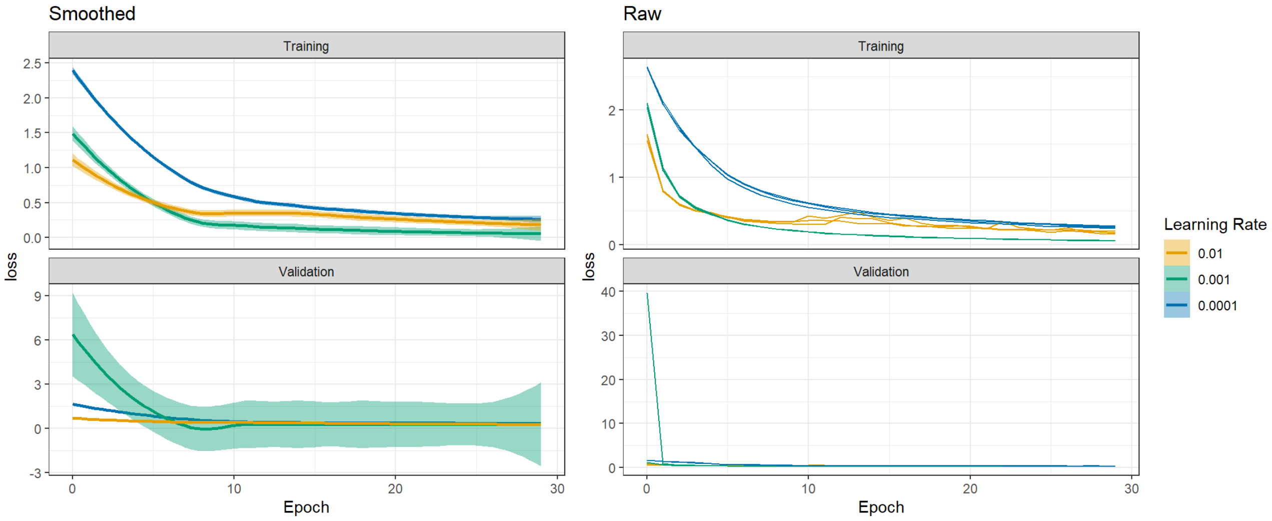
Smoothed
Raw
Training
Validation
loss
Epoch
Learning Rate
0.01
0.001
0.0001

B

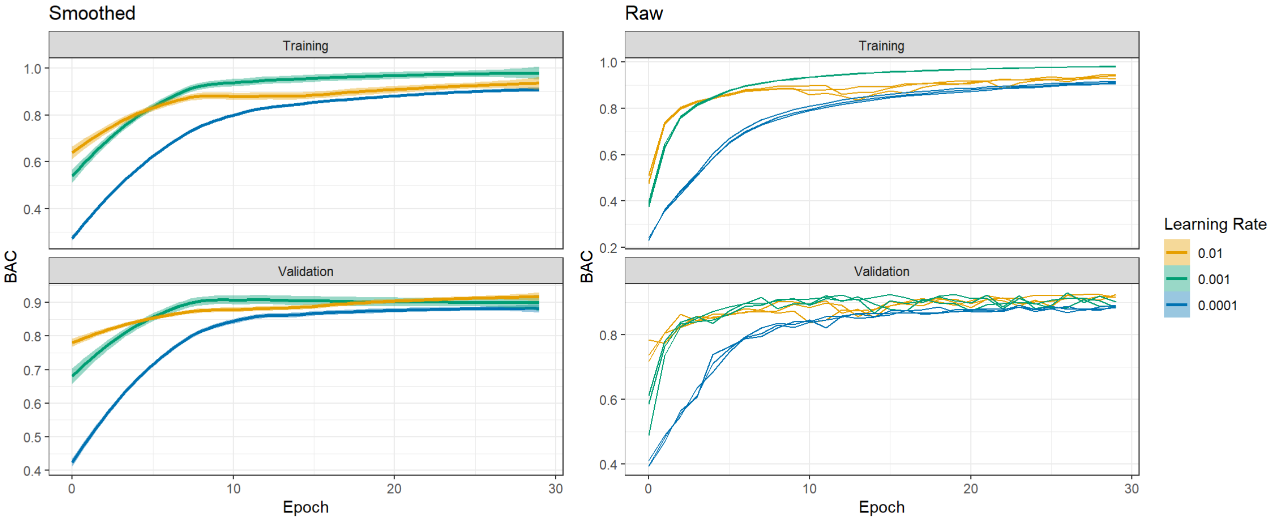
Smoothed
Raw
Training
Validation
BAC
Epoch
Learning Rate
0.01
0.001
0.0001

C

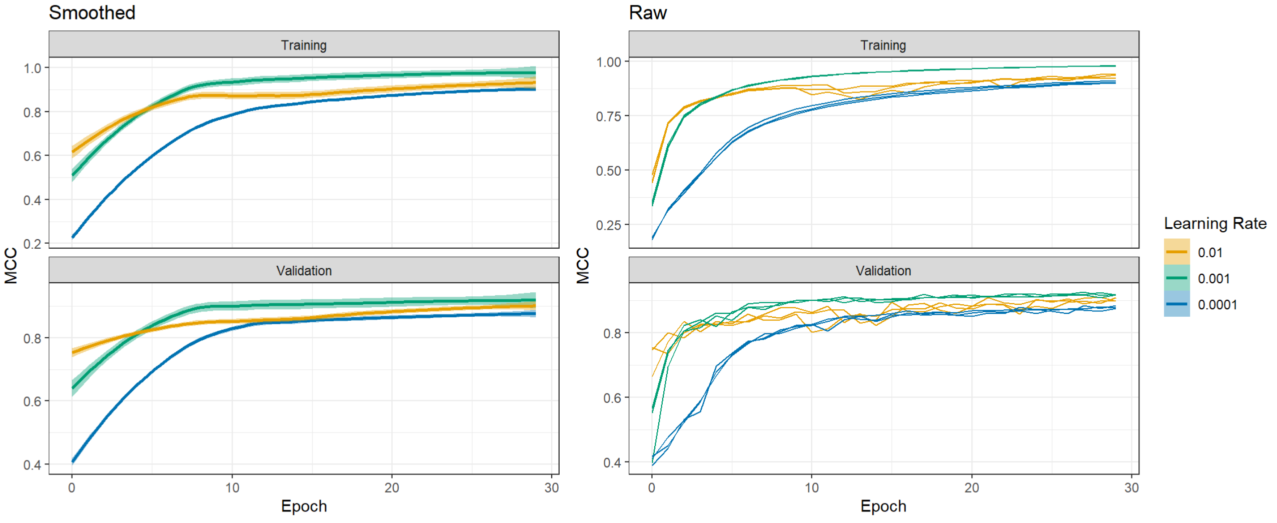

Supplemental Figure 2: Learning rate scheduler. Cross entropy loss (A), balanced accuracy (B) and Matthews correlation coefficient (C) for training and validation data across all training epochs. The plots on the left smooth all values for the three different iterations, while the plots on the right show the raw values.

A

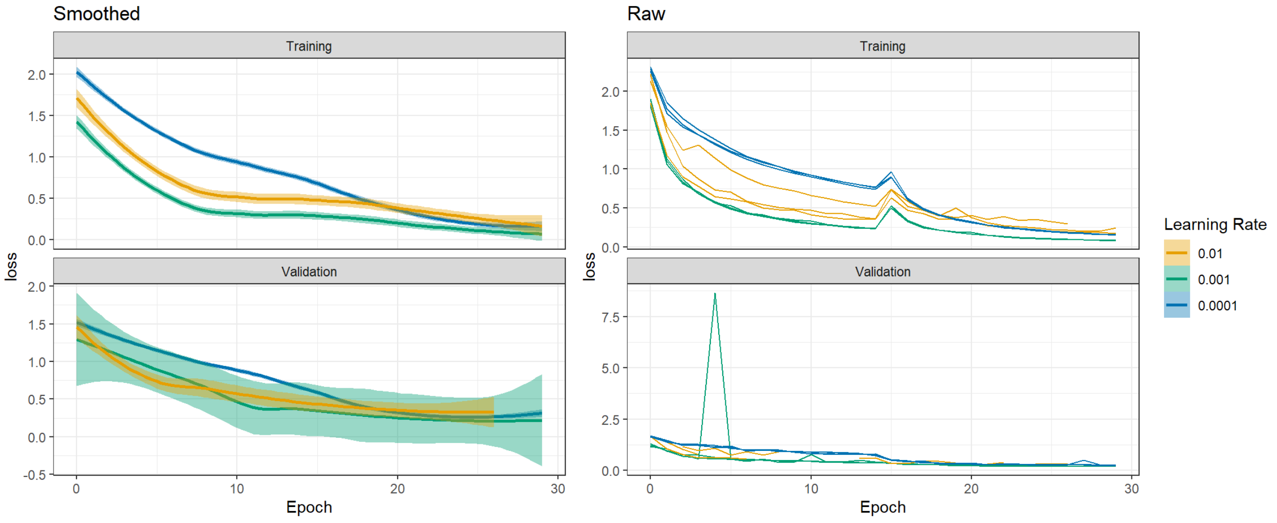
Smoothed
Raw
Training
Validation
loss
Epoch
Learning Rate
0.01
0.001
0.0001

B

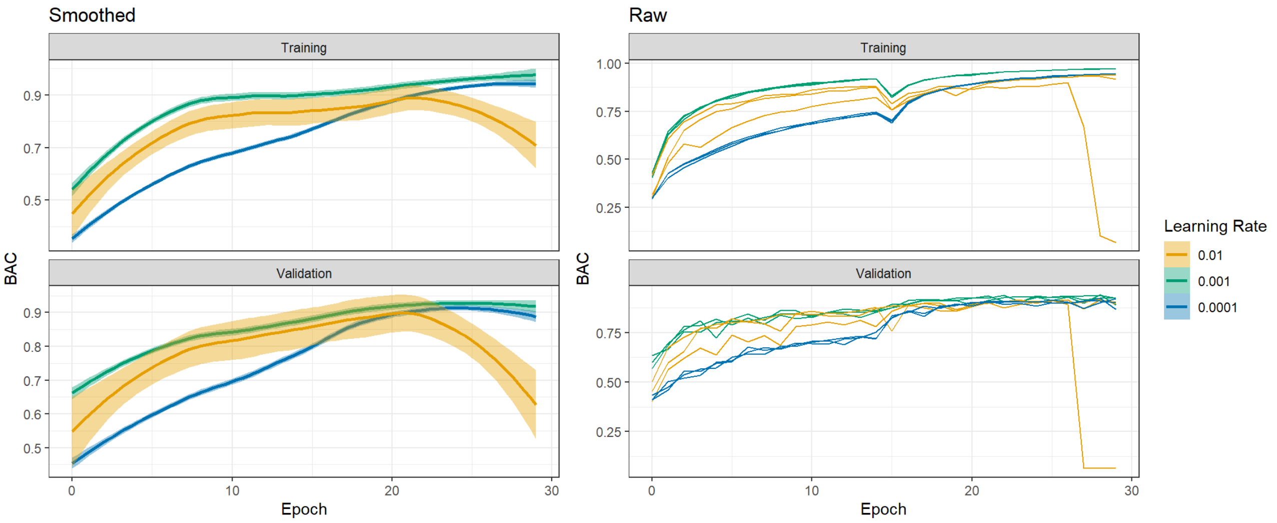
Smoothed
Raw
Training
Validation
BAC
Epoch
Learning Rate
0.01
0.001
0.0001

C

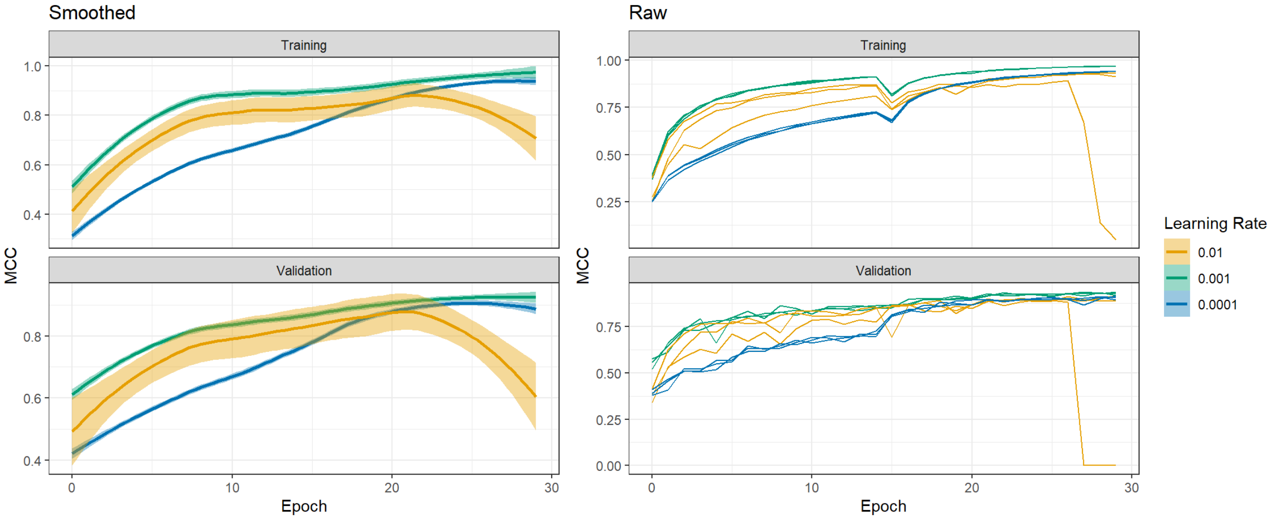

Supplemental Figure 3: Progressive learning. Cross entropy loss (A), balanced accuracy (B) and Matthews correlation coefficient (C) for training and validation data across all training epochs. The plots on the left smooth all values for the three different iterations, while the plots on the right show the raw values.

A

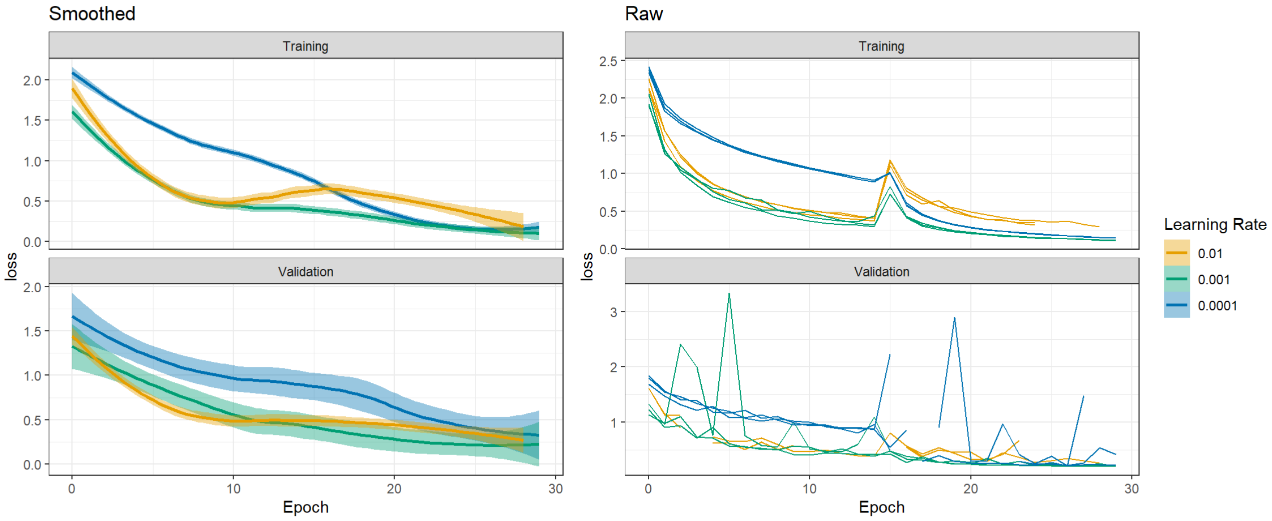
Smoothed
Raw
Training
Validation
loss
Epoch
Learning Rate
0.01
0.001
0.0001

B

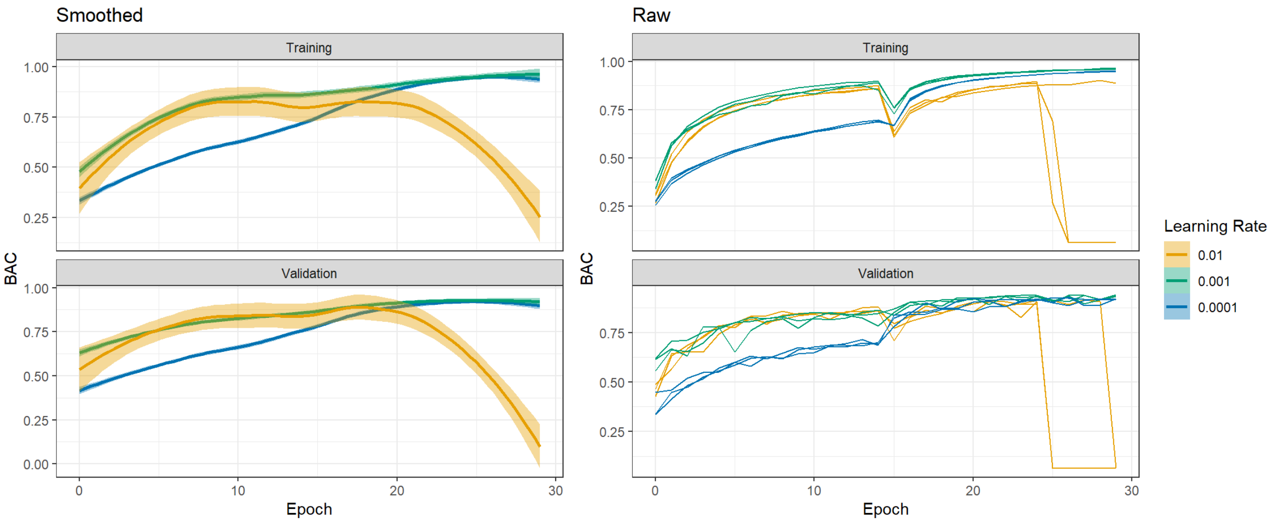
Smoothed
Raw
Training
Validation
BAC
Epoch
Learning Rate
0.01
0.001
0.0001

C

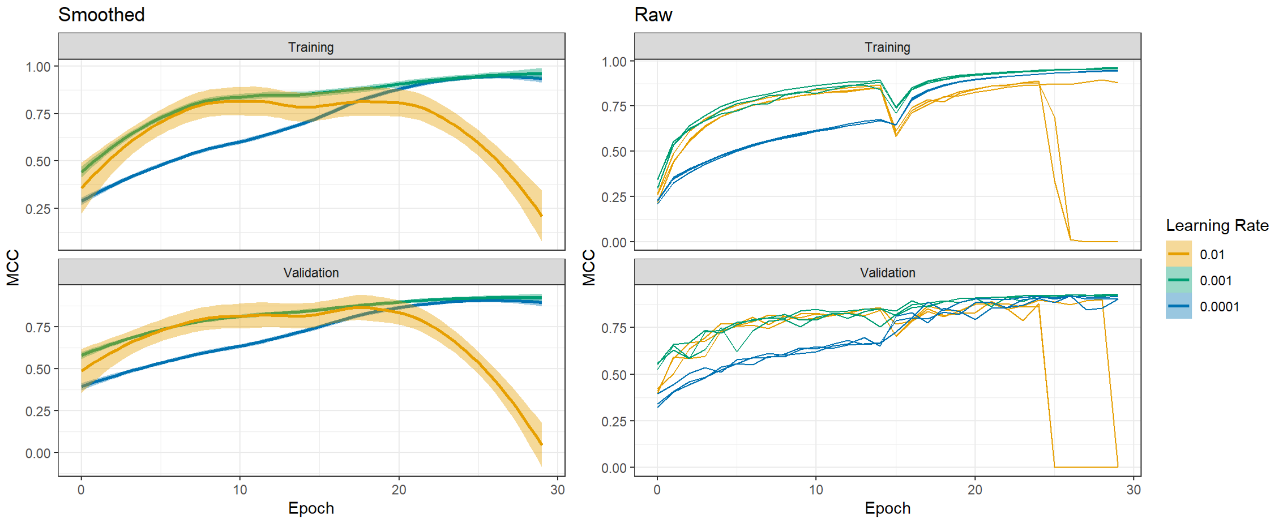

Supplemental Figure 4: Larger model. Cross entropy loss (A), balanced accuracy (B) and Matthews correlation coefficient (C) for training and validation data across all training epochs. The plots on the left smooth all values for the three different iterations, while the plots on the right show the raw values.

A

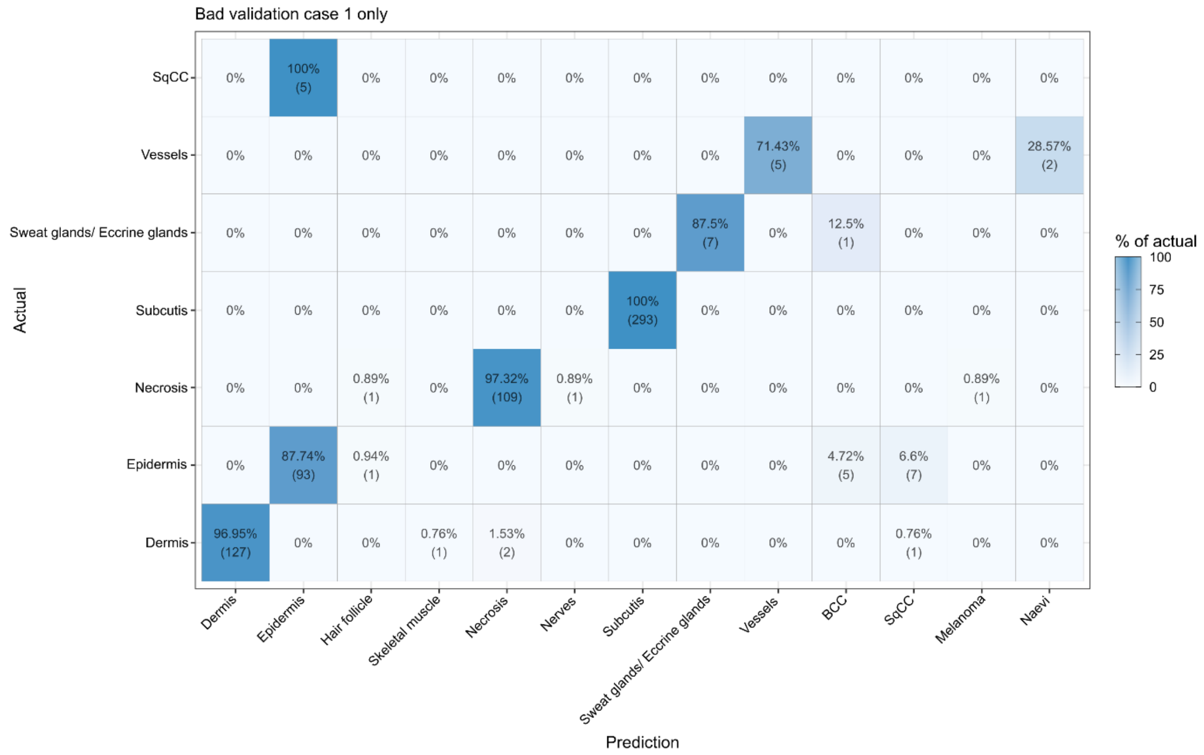

B

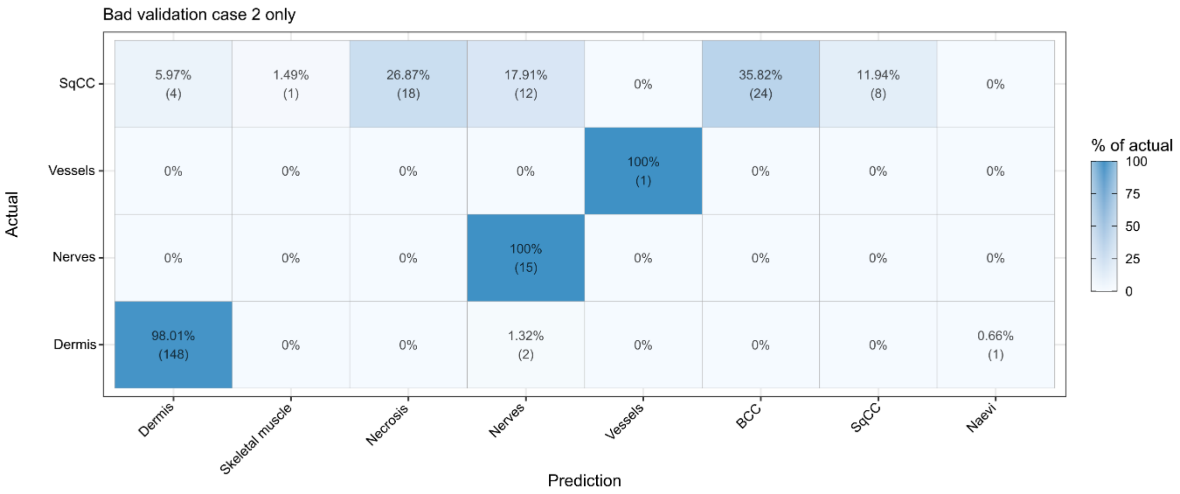

Supplemental Figure 5: Two cases with a proportion of tumor image tiles with <25 % being in the correct tumor category in the validation set. Case 1 (A) shows clear misclassifications. This case had a very low number of annotated tumor tiles (n = 5). As many of the non-tumor tiles were misclassified as the correct tumor category, this case would overall have had the correct tumor prediction on the case level, based on a majority vote. Case 2 (B) shows a high rate of misclassifications with basal cell carcinoma. BCC: basal cell carcinoma, SqCC: squamous cell carcinoma.

A

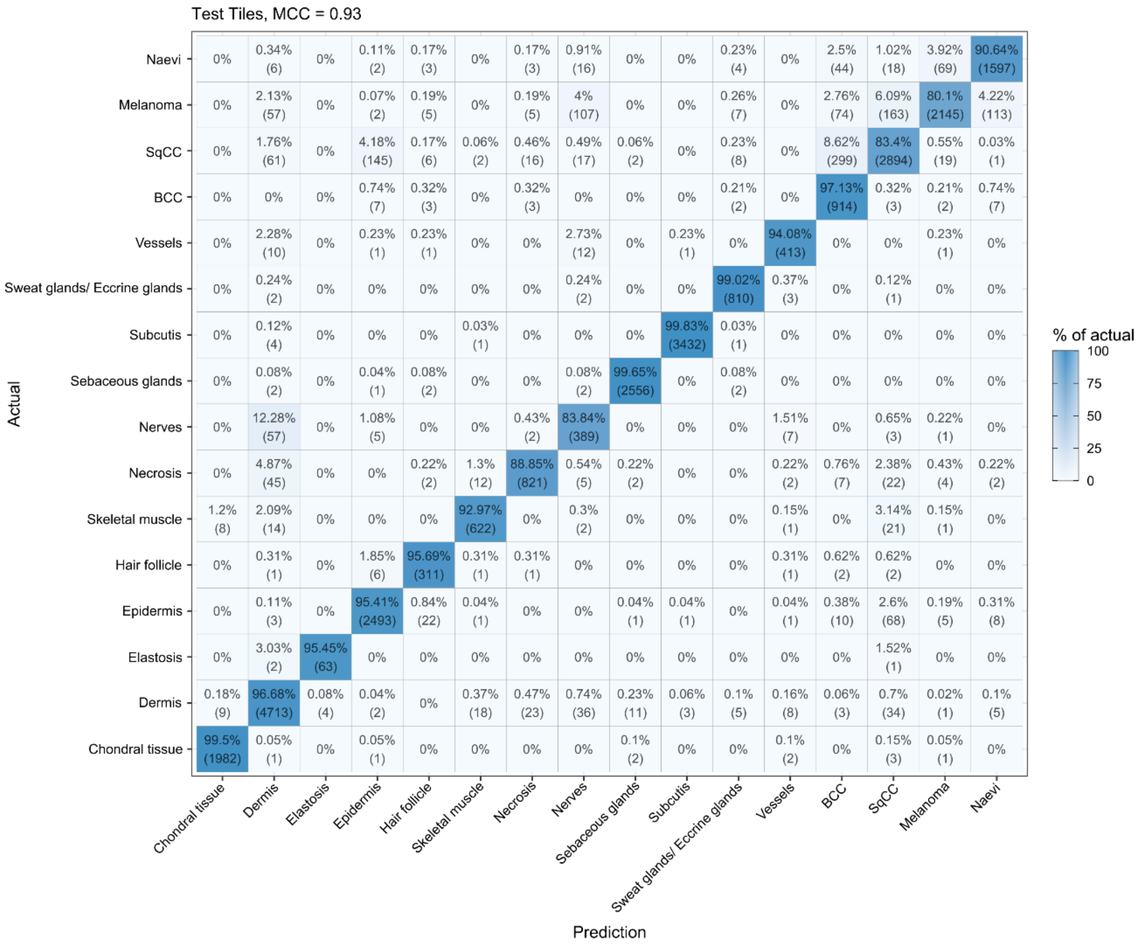
Test Tiles, MCC = 0.93
Actual
Prediction
% of actual
100
75
50
25
0
Naevi: 0% | 0.34% (6) | 0% | 0.11% (2) | 0.17% (3) | 0% | 0.17% (3) | 0.91% (16) | 0% | 0% | 0.23% (4) | 0% | 2.5% (44) | 1.02% (18) | 3.92% (69) | 90.64% (1597)
Melanoma: 0% | 2.13% (57) | 0% | 0.07% (2) | 0.19% (5) | 0% | 0.19% (5) | 4% (107) | 0% | 0% | 0.26% (7) | 0% | 2.76% (74) | 6.09% (163) | 80.1% (2145) | 4.22% (113)
SqCC: 0% | 1.76% (61) | 0% | 4.18% (145) | 0.17% (6) | 0.06% (2) | 0.46% (16) | 0.49% (17) | 0.06% (2) | 0% | 0.23% (8) | 0% | 8.62% (299) | 83.4% (2894) | 0.55% (19) | 0.03% (1)
BCC: 0% | 0% | 0% | 0.74% (7) | 0.32% (3) | 0% | 0.32% (3) | 0% | 0% | 0% | 0.21% (2) | 0% | 97.13% (914) | 0.32% (3) | 0.21% (2) | 0.74% (7)
Vessels: 0% | 2.28% (10) | 0% | 0.23% (1) | 0.23% (1) | 0% | 0% | 2.73% (12) | 0% | 0.23% (1) | 0% | 94.08% (413) | 0% | 0% | 0.23% (1) | 0%
Sweat glands/ Eccrine glands: 0% | 0.24% (2) | 0% | 0% | 0% | 0% | 0% | 0.24% (2) | 0% | 0% | 99.02% (810) | 0.37% (3) | 0% | 0.12% (1) | 0% | 0%
Subcutis: 0% | 0.12% (4) | 0% | 0% | 0% | 0.03% (1) | 0% | 0% | 0% | 99.83% (3432) | 0.03% (1) | 0% | 0% | 0% | 0% | 0%
Sebaceous glands: 0% | 0.08% (2) | 0% | 0.04% (1) | 0.08% (2) | 0% | 0% | 0.08% (2) | 99.65% (2556) | 0% | 0.08% (2) | 0% | 0% | 0% | 0% | 0%
Nerves: 0% | 12.28% (57) | 0% | 1.08% (5) | 0% | 0% | 0.43% (2) | 83.84% (389) | 0% | 0% | 0% | 1.51% (7) | 0% | 0.65% (3) | 0.22% (1) | 0%
Necrosis: 0% | 4.87% (45) | 0% | 0% | 0.22% (2) | 1.3% (12) | 88.85% (821) | 0.54% (5) | 0.22% (2) | 0% | 0% | 0.22% (2) | 0.76% (7) | 2.38% (22) | 0.43% (4) | 0.22% (2)
Skeletal muscle: 1.2% (8) | 2.09% (14) | 0% | 0% | 0% | 92.97% (622) | 0% | 0.3% (2) | 0% | 0% | 0% | 0.15% (1) | 0% | 3.14% (21) | 0.15% (1) | 0%
Hair follicle: 0% | 0.31% (1) | 0% | 1.85% (6) | 95.69% (311) | 0.31% (1) | 0.31% (1) | 0% | 0% | 0% | 0% | 0.31% (1) | 0.62% (2) | 0.62% (2) | 0% | 0%
Epidermis: 0% | 0.11% (3) | 0% | 95.41% (2493) | 0.84% (22) | 0.04% (1) | 0% | 0% | 0.04% (1) | 0.04% (1) | 0% | 0.04% (1) | 0.38% (10) | 2.6% (68) | 0.19% (5) | 0.31% (8)
Elastosis: 0% | 3.03% (2) | 95.45% (63) | 0% | 0% | 0% | 0% | 0% | 0% | 0% | 0% | 0% | 0% | 1.52% (1) | 0% | 0%
Dermis: 0.18% (9) | 96.68% (4713) | 0.08% (4) | 0.04% (2) | 0% | 0.37% (18) | 0.47% (23) | 0.74% (36) | 0.23% (11) | 0.06% (3) | 0.1% (5) | 0.16% (8) | 0.06% (3) | 0.7% (34) | 0.02% (1) | 0.1% (5)
Chondral tissue: 99.5% (1982) | 0.05% (1) | 0% | 0.05% (1) | 0% | 0% | 0% | 0% | 0.1% (2) | 0% | 0% | 0.1% (2) | 0% | 0.15% (3) | 0.05% (1) | 0%
Chondral tissue
Dermis
Elastosis
Epidermis
Hair follicle
Skeletal muscle
Necrosis
Nerves
Sebaceous glands
Subcutis
Sweat glands/ Eccrine glands
Vessels
BCC
SqCC
Melanoma
Naevi

B

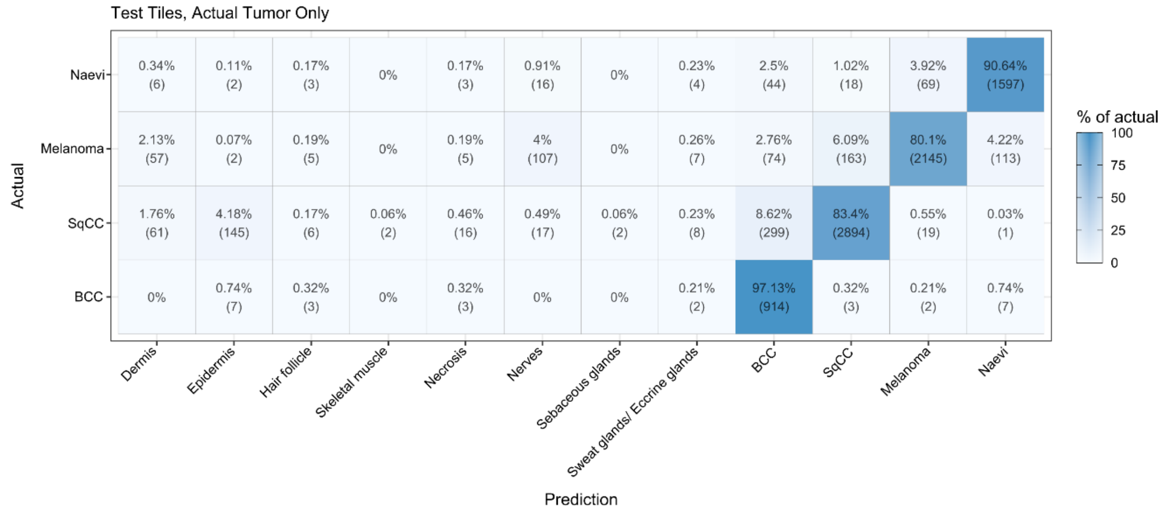
Test Tiles, Actual Tumor Only
Actual
Prediction
% of actual
100
75
50
25
0
Naevi: 0.34% (6) | 0.11% (2) | 0.17% (3) | 0% | 0.17% (3) | 0.91% (16) | 0% | 0.23% (4) | 2.5% (44) | 1.02% (18) | 3.92% (69) | 90.64% (1597)
Melanoma: 2.13% (57) | 0.07% (2) | 0.19% (5) | 0% | 0.19% (5) | 4% (107) | 0% | 0.26% (7) | 2.76% (74) | 6.09% (163) | 80.1% (2145) | 4.22% (113)
SqCC: 1.76% (61) | 4.18% (145) | 0.17% (6) | 0.06% (2) | 0.46% (16) | 0.49% (17) | 0.06% (2) | 0.23% (8) | 8.62% (299) | 83.4% (2894) | 0.55% (19) | 0.03% (1)
BCC: 0% | 0.74% (7) | 0.32% (3) | 0% | 0.32% (3) | 0% | 0% | 0.21% (2) | 97.13% (914) | 0.32% (3) | 0.21% (2) | 0.74% (7)
Dermis
Epidermis
Hair follicle
Skeletal muscle
Necrosis
Nerves
Sebaceous glands
Sweat glands/ Eccrine glands
BCC
SqCC
Melanoma
Naevi

C

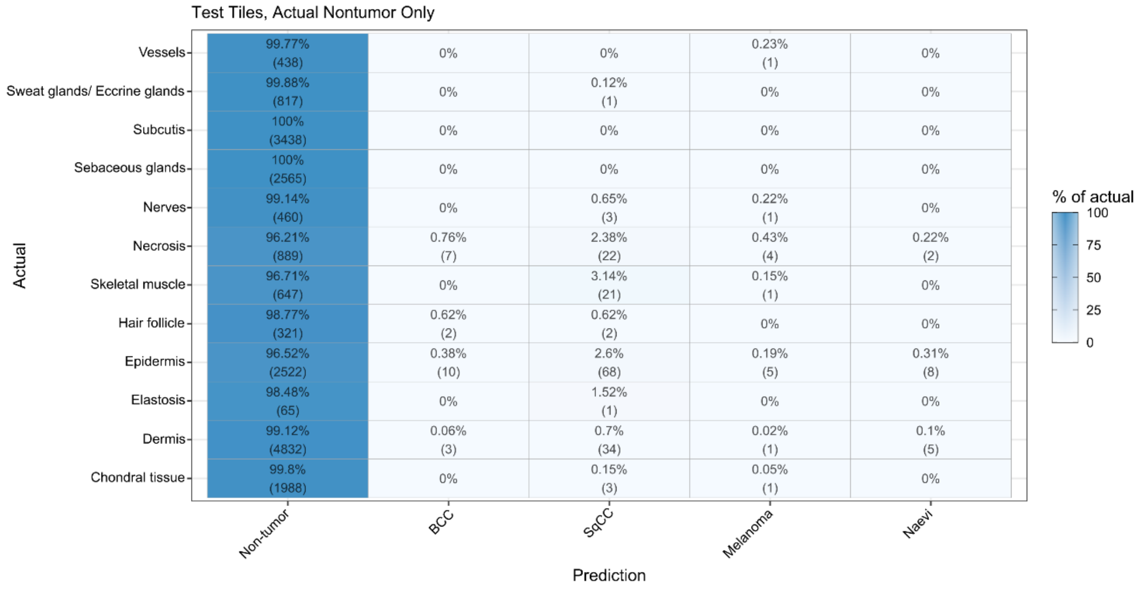

Supplemental Figure 6: Confusion matrix on the test set based on image tiles. High concordance between actual and predicted classes can be observed. The algorithm shows a higher rate of misclassification of nerves with dermis, which can be explained since nerves are commonly observed in the dermis (A). In tumor categories (B) a higher rate of misclassifications was observed for squamous cell carcinomas that were predicted as basal cell carcinoma. The misclassification of non-tumor categories as tumor was rare but observed most with skeletal muscle, misclassified as squamous cell carcinoma (C). BCC: basal cell carcinoma, SqCC: squamous cell carcinoma.

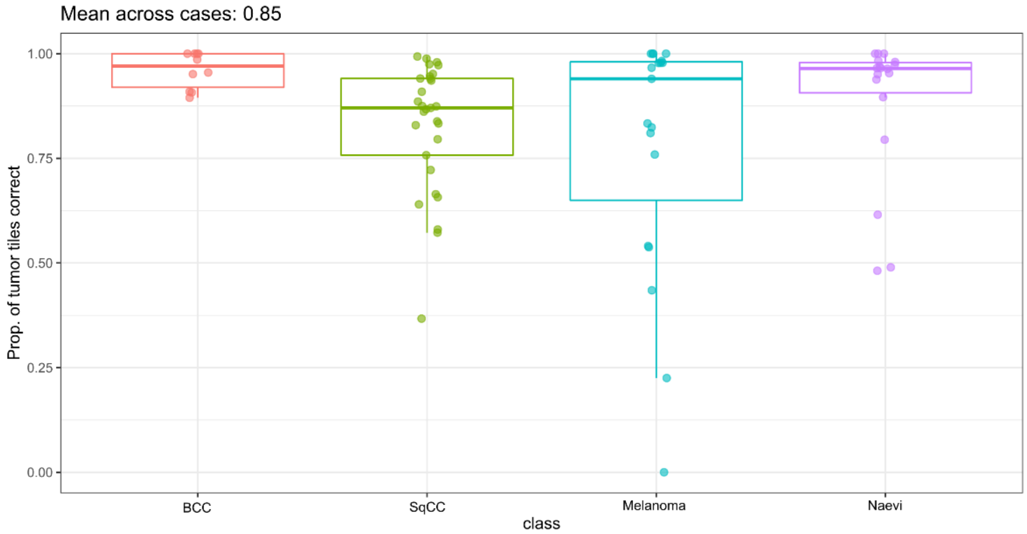

Supplemental Figure 7: Proportion of tumor image tiles that was correctly classified on patient level in the test set.
Most tumors were correctly classified on patient level. Two patients with melanoma were misclassified. BCC: basal cell carcinoma, SqCC: squamous cell carcinoma.

A

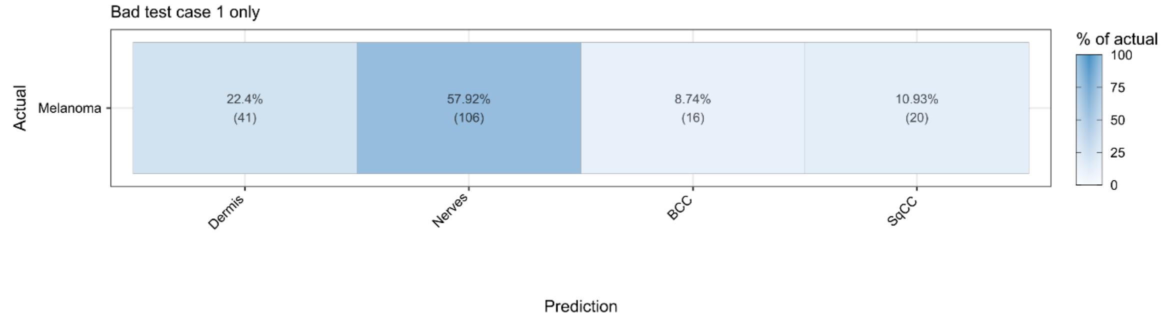

B

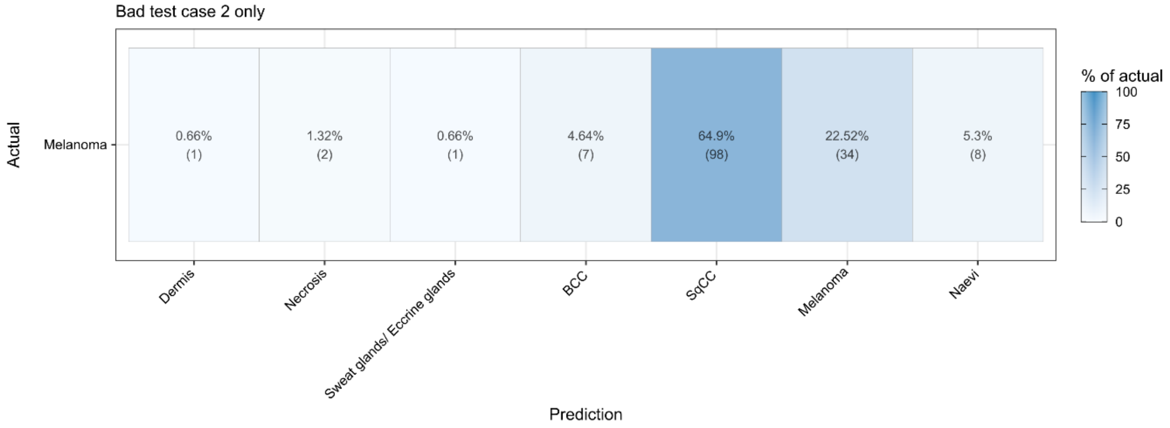

Supplemental Figure 8: Two cases with a proportion of tumor image tiles with <25 % being in the correct tumor category in the test set.
Case 1 (A) shows clear misclassifications of a melanoma as basal cell carcinoma and squamous cell carcinoma, respectively. Case 2 (B) shows a high rate of misclassifications with basal cell carcinoma, followed by prediction as melanoma. BCC: basal cell carcinoma, SqCC: squamous cell carcinoma.

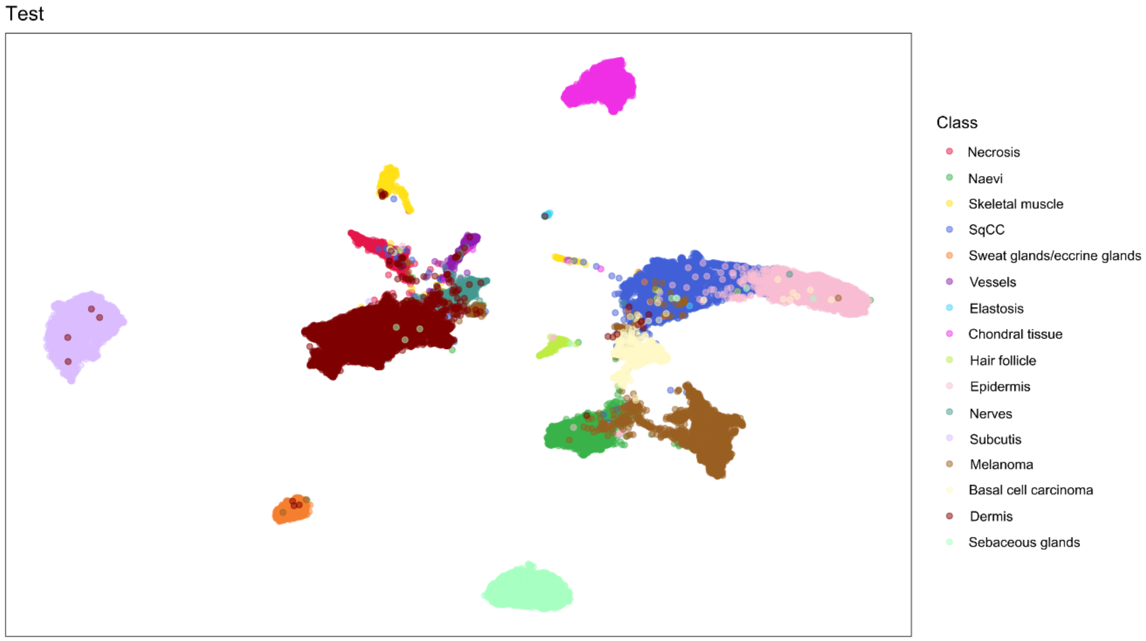

Supplemental Figure 9: Dimension reduction using uniform manifold approximation and projection based on the last convolutional layer after the last pooling operation of the test data. Proximity of image classes that resemble each other morphologically such as melanocytic tumors can be observed. On the other hand, image categories that are morphologically very different such as skeletal muscle, sebaceous glands or chrondral tissue show distinct clusters. BCC: basal cell carcinoma, SqCC: squamous cell carcinoma.

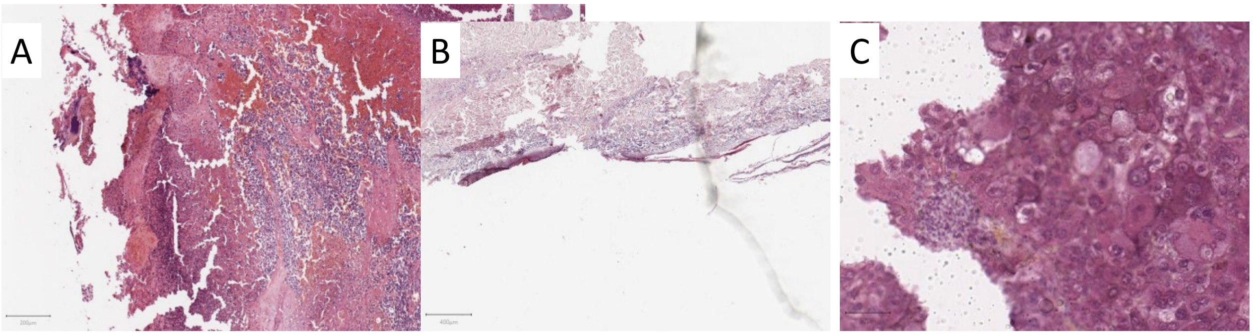

Supplemental Figure 10: Examples of quality issues of the external test set. Tissue crackles and hemorrhage (A), foreign material (B), blurry and thick tissue areas (C) which severely alter cytomorphological assessment and are suboptimal for automated classification by deep learning.
